# 2,4-Dichloro-*N*-(4-methyl­phen­yl)benzene­sulfonamide

**DOI:** 10.1107/S1600536809053707

**Published:** 2009-12-19

**Authors:** B. Thimme Gowda, Sabine Foro, P. G. Nirmala, Hartmut Fuess

**Affiliations:** a Department of Chemistry, Mangalore University, Mangalagangotri 574 199, Mangalore, India; b Institute of Materials Science, Darmstadt University of Technology, Petersenstrasse 23, D-64287 Darmstadt, Germany

## Abstract

The title compound, C_13_H_11_Cl_2_NO_2_S, crystallizes with four independent mol­ecules in the asymmetric unit. In each of the four mol­ecules, the conformation of the N—C bond in the C—SO_2_—NH—C segment is *gauche* with respect to both S=O bonds. The mol­ecules are twisted at the S—N bonds with C—SO_2_—NH—C torsion angles of 60.6 (4), −59.7 (3), 63.9 (4) and 53.0 (4)°. The benzene rings in two of the mol­ecules are disordered with multiple positions resolved in each case. The crystal structure features inversion dimers linked by pairs of N—H⋯O hydrogen bonds for each of the four molecules.

## Related literature

For the preparation of the title compound, see: Savitha & Gowda (2006 ▶). For our studies of the effect of substituents on the structures of *N*-(ar­yl)aryl­sulfonamides, see: Gowda *et al.* (2009**a* ▶,b* ▶). For related structures, see: Gelbrich *et al.* (2007 ▶); Perlovich *et al.* (2006 ▶).
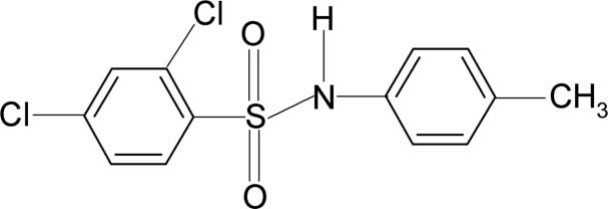


## Experimental

### 

#### Crystal data

C_13_H_11_Cl_2_NO_2_S
*M* *_r_* = 316.19
Triclinic, 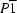

*a* = 11.760 (2) Å
*b* = 14.875 (2) Å
*c* = 16.672 (3) Å
α = 85.06 (1)°
β = 75.26 (1)°
γ = 87.20 (1)°
*V* = 2808.9 (8) Å^3^
*Z* = 8
Cu *K*α radiation
μ = 5.53 mm^−1^
*T* = 299 K
0.50 × 0.35 × 0.30 mm

#### Data collection

Enraf–Nonius CAD-4 diffractometer
Absorption correction: ψ scan (North *et al.*, 1968 ▶) *T*_min_ = 0.169, *T*_max_ = 0.288
10777 measured reflections
9992 independent reflections
7795 reflections with *I* > 2σ(*I*)
*R*_int_ = 0.016
3 standard reflections every 3 min
intensity decay: 1.0%

#### Refinement

*R*[*F* ^2^ > 2σ(*F* ^2^)] = 0.062
*wR*(*F* ^2^) = 0.199
*S* = 1.05
9992 reflections
802 parameters
18 restraints
H atoms treated by a mixture of independent and constrained refinement
Δρ_max_ = 0.70 e Å^−3^
Δρ_min_ = −0.67 e Å^−3^

### 

Data collection: *CAD-4-PC* (Enraf–Nonius, 1996 ▶); cell refinement: *CAD-4-PC*; data reduction: *REDU4* (Stoe & Cie, 1987 ▶); program(s) used to solve structure: *SHELXS97* (Sheldrick, 2008 ▶); program(s) used to refine structure: *SHELXL97* (Sheldrick, 2008 ▶); molecular graphics: *PLATON* (Spek, 2009 ▶); software used to prepare material for publication: *SHELXL97*.

## Supplementary Material

Crystal structure: contains datablocks I, global. DOI: 10.1107/S1600536809053707/tk2592sup1.cif

Structure factors: contains datablocks I. DOI: 10.1107/S1600536809053707/tk2592Isup2.hkl

Additional supplementary materials:  crystallographic information; 3D view; checkCIF report

## Figures and Tables

**Table 1:** Hydrogen-bond geometry (Å, °)

*D*—H⋯*A*	*D*—H	H⋯*A*	*D*⋯*A*	*D*—H⋯*A*
N1—H1*N*⋯O2^i^	0.83 (3)	2.26 (3)	3.079 (4)	169 (5)
N2—H2*N*⋯O3^ii^	0.85 (3)	2.24 (3)	3.080 (4)	169 (4)
N3—H3*N*⋯O5^iii^	0.82 (3)	2.14 (3)	2.956 (5)	178 (5)
N4—H4*N*⋯O8^iv^	0.86 (3)	2.37 (3)	3.226 (5)	177 (4)

Symmetry codes: (i) 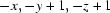; (ii) 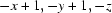; (iii) 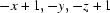; (iv) 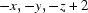.

**Fig. 1.**
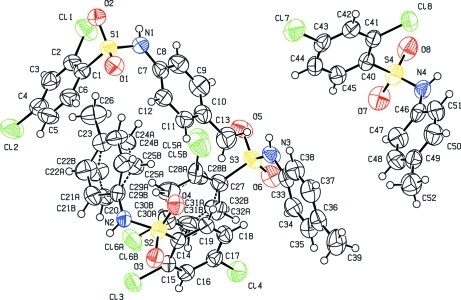
Molecular structure of (I), showing the atom labeling and displacement ellipsoids drawn at the 50% probability level. Both disorder components are shown. The disorder components are shown with dashed bonds.

**Fig. 2.**
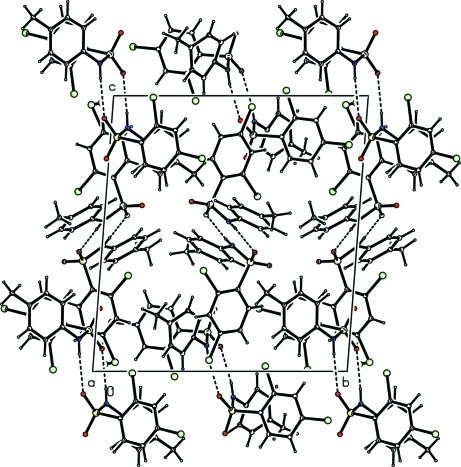
Molecular packing of (I) with hydrogen bonding shown as dashed lines. For more clarity the disorder components were omitted.

## supplementary crystallographic information

### Comment

In the present work, as part of a study of substituent effects on the structures of *N*-(aryl)arylsulfonamides (Gowda *et al.*, 2009*a, b*), the structure of 2,4-dichloro-*N*-(4-methylphenyl)benzenesulfonamide (I) has been determined. The asymmetric unit of (I) contains four independent molecules. The conformations of the N—C bonds in the C—SO_2_—NH—C segment have *gauche* torsions with respect to the S═O bonds (Fig. 1). The molecules are twisted at the S–N bonds with the C—SO_2_—NH—C torsion angles being 60.6 (4)°, -59.7 (3)°, 63.9 (4)°, and 53.0 (4)°, respectively, compared to the values of -48.2 (2)° in 2,4-dichloro-*N*-(3,4-dichlorophenyl)benzenesulfonamide (II) (Gowda *et al.*, 2009*b*) and -69.7 (2)° in 2,4-dimethyl-*N*-(3,4-dichlorophenyl)benzenesulfonamide (III) (Gowda *et al.*, 2009*a*).

The sulfonyl benzene and the aniline benzene rings in the four molecules of (I) are tilted relative to each other by 85.2 (1)° (molecule 1), 80.5 (2)° (molecule 2, disordered orientation A), 80.1 (2)° (molecule 2, orientation B), 87.5 (7) (molecule 3, disordered orientation A), 87.0 (6)° (molecule 3, orientation B) and 72.4 (1)° (molecule 4), compared to the values of 68.9 (1)° in (II) and 82.4 (1)° in (III). The other bond parameters in (I) are similar to those observed in (II), (III) and other aryl sulfonamides (Perlovich *et al.*, 2006; Gelbrich *et al.*, 2007).

In the crystal structure, the pairs of intermolecular N–H···O hydrogen bonds link the molecules through inversion-related dimers, Fig. 2 and Table 1.

### Experimental

The solution of 1,3-dichlorobenzene (10 ml) in chloroform (40 ml) was treated drop-wise with chlorosulfonic acid (25 ml) at 273 K. After the initial evolution of hydrogen chloride subsided, the reaction mixture was brought to room temperature and poured into crushed ice in a beaker. The chloroform layer was separated, washed with cold water and allowed to evaporate slowly. The residual 2,4-dichlorobenzenesulfonylchloride was treated with a stoichiometric amount of *p*-toluidine and boiled for ten minutes. The reaction mixture was then cooled to room temperature and added to ice cold water (100 ml). The resultant solid 2,4-dichloro-*N*-(4-methylphenyl)benzenesulfonamide was filtered under suction and washed thoroughly with cold water. It was then recrystallized to constant melting point from dilute ethanol. The purity of the compound was checked and characterized by recording its infrared and NMR spectra (Savitha & Gowda, 2006). The single crystals used in X-ray diffraction studies were grown in ethanolic solution by slow evaporation at room temperature.

### Refinement

The H atoms of the NH groups were located in a difference map and refined with the distance restraint N—H = 0.86 (3) Å. The other H atoms were positioned with idealized geometry using a riding model [C—H = 0.93–0.96 Å]. All H atoms were refined with isotropic displacement parameters (set to 1.2 times of the *U*_eq_ of the parent atom).

Two of the benzene rings with C21, C22, C24, C25 and C28, C29, C30, C31, C32, respectively, with Cl5 and Cl6 are disordered and were refined using a split model. The corresponding site-occupation factors were fixed to 0.50:0.50, and their corresponding bond distances in the disordered groups were restrained to be equal.

### Crystal data

**Table:**

C_13_H_11_Cl_2_NO_2_S	*Z* = 8
*M**_r_* = 316.19	*F*(000) = 1296
Triclinic, *P*1	*D*_x_ = 1.495 Mg m^−^^3^
Hall symbol: -P 1	Cu *K*α radiation, λ = 1.54180 Å
*a* = 11.760 (2) Å	Cell parameters from 25 reflections
*b* = 14.875 (2) Å	θ = 5.3–17.4°
*c* = 16.672 (3) Å	µ = 5.53 mm^−^^1^
α = 85.06 (1)°	*T* = 299 K
β = 75.26 (1)°	Rod, colourless
γ = 87.20 (1)°	0.50 × 0.35 × 0.30 mm
*V* = 2808.9 (8) Å^3^	

### Data collection

**Table:**

Enraf–Nonius CAD-4 diffractometer	7795 reflections with *I* > 2σ(*I*)
Radiation source: fine-focus sealed tube	*R*_int_ = 0.016
graphite	θ_max_ = 67.0°, θ_min_ = 2.8°
ω/2θ scans	*h* = −14→13
Absorption correction: ψ scan (North *et al.*, 1968)	*k* = −17→17
*T*_min_ = 0.169, *T*_max_ = 0.288	*l* = −19→1
10777 measured reflections	3 standard reflections every 120 min
9992 independent reflections	intensity decay: 1.0%

### Refinement

**Table:**

Refinement on *F*^2^	Secondary atom site location: difference Fourier map
Least-squares matrix: full	Hydrogen site location: inferred from neighbouring sites
*R*[*F*^2^ > 2σ(*F*^2^)] = 0.062	H atoms treated by a mixture of independent and constrained refinement
*wR*(*F*^2^) = 0.199	*w* = 1/[σ^2^(*F*_o_^2^) + (0.1384*P*)^2^ + 0.7496*P*] where *P* = (*F*_o_^2^ + 2*F*_c_^2^)/3
*S* = 1.05	(Δ/σ)_max_ = 0.003
9992 reflections	Δρ_max_ = 0.70 e Å^−^^3^
802 parameters	Δρ_min_ = −0.67 e Å^−^^3^
18 restraints	Extinction correction: *SHELXL97* (Sheldrick, 2008), Fc^*^=kFc[1+0.001xFc^2^λ^3^/sin(2θ)]^-1/4^
Primary atom site location: structure-invariant direct methods	Extinction coefficient: 0.0029 (3)

### Special details

**Table:**

Geometry. All e.s.d.'s (except the e.s.d. in the dihedral angle between two l.s. planes) are estimated using the full covariance matrix. The cell e.s.d.'s are taken into account individually in the estimation of e.s.d.'s in distances, angles and torsion angles; correlations between e.s.d.'s in cell parameters are only used when they are defined by crystal symmetry. An approximate (isotropic) treatment of cell e.s.d.'s is used for estimating e.s.d.'s involving l.s. planes.
Refinement. Refinement of *F*^2^ against ALL reflections. The weighted *R*-factor *wR* and goodness of fit *S* are based on *F*^2^, conventional *R*-factors *R* are based on *F*, with *F* set to zero for negative *F*^2^. The threshold expression of *F*^2^ > σ(*F*^2^) is used only for calculating *R*-factors(gt) *etc*. and is not relevant to the choice of reflections for refinement. *R*-factors based on *F*^2^ are statistically about twice as large as those based on *F*, and *R*- factors based on ALL data will be even larger.

### Fractional atomic coordinates and isotropic or equivalent isotropic displacement parameters (Å^2^)

**Table:**

	*x*	*y*	*z*	*U*_iso_*/*U*_eq_	Occ. (<1)
Cl1	−0.08874 (12)	0.59478 (9)	0.63447 (7)	0.0872 (4)	
Cl2	−0.12670 (14)	0.54619 (13)	0.95925 (8)	0.1092 (5)	
S1	0.07830 (8)	0.41228 (7)	0.60370 (6)	0.0560 (2)	
O1	0.1536 (3)	0.33756 (19)	0.61595 (18)	0.0668 (7)	
O2	−0.0180 (3)	0.3988 (2)	0.56828 (18)	0.0674 (7)	
N1	0.1532 (3)	0.4898 (3)	0.5437 (2)	0.0609 (8)	
H1N	0.108 (4)	0.518 (3)	0.519 (3)	0.073*	
C1	0.0217 (3)	0.4545 (3)	0.7027 (2)	0.0545 (8)	
C2	−0.0507 (3)	0.5305 (3)	0.7158 (2)	0.0592 (9)	
C3	−0.0953 (4)	0.5585 (3)	0.7950 (3)	0.0694 (11)	
H3	−0.1433	0.6101	0.8030	0.083*	
C4	−0.0686 (4)	0.5099 (4)	0.8609 (3)	0.0725 (12)	
C5	0.0029 (4)	0.4340 (4)	0.8506 (3)	0.0771 (13)	
H5	0.0216	0.4016	0.8960	0.092*	
C6	0.0466 (4)	0.4068 (3)	0.7712 (3)	0.0680 (10)	
H6	0.0941	0.3548	0.7637	0.082*	
C7	0.2553 (3)	0.5316 (2)	0.5541 (2)	0.0532 (8)	
C8	0.2770 (4)	0.6176 (3)	0.5178 (3)	0.0695 (11)	
H8	0.2245	0.6471	0.4904	0.083*	
C9	0.3769 (5)	0.6602 (3)	0.5220 (3)	0.0752 (12)	
H9	0.3913	0.7182	0.4968	0.090*	
C10	0.4551 (4)	0.6186 (3)	0.5628 (2)	0.0625 (10)	
C11	0.4318 (4)	0.5330 (3)	0.5987 (2)	0.0612 (9)	
H11	0.4840	0.5036	0.6265	0.073*	
C12	0.3323 (3)	0.4890 (3)	0.5948 (2)	0.0592 (9)	
H12	0.3181	0.4309	0.6197	0.071*	
C13	0.5661 (5)	0.6658 (4)	0.5654 (4)	0.0893 (15)	
H13A	0.5472	0.7278	0.5764	0.107*	
H13B	0.6229	0.6630	0.5129	0.107*	
H13C	0.5983	0.6363	0.6087	0.107*	
Cl3	0.46567 (10)	0.32311 (8)	−0.01888 (6)	0.0732 (3)	
Cl4	0.22211 (10)	0.06126 (7)	0.17786 (7)	0.0745 (3)	
S2	0.44663 (7)	0.43952 (6)	0.14212 (6)	0.0506 (2)	
O3	0.3878 (2)	0.49470 (18)	0.09004 (18)	0.0640 (7)	
O4	0.4332 (2)	0.46087 (18)	0.22529 (17)	0.0633 (7)	
N2	0.5839 (3)	0.4397 (2)	0.09413 (19)	0.0511 (7)	
H2N	0.599 (4)	0.452 (3)	0.0419 (17)	0.061*	
C14	0.3966 (3)	0.3273 (2)	0.1493 (2)	0.0471 (7)	
C15	0.3988 (3)	0.2804 (2)	0.0807 (2)	0.0520 (8)	
C16	0.3475 (3)	0.1974 (3)	0.0899 (2)	0.0573 (9)	
H16	0.3502	0.1658	0.0436	0.069*	
C17	0.2922 (3)	0.1620 (2)	0.1688 (2)	0.0545 (8)	
C18	0.2910 (3)	0.2054 (3)	0.2379 (2)	0.0575 (9)	
H18	0.2552	0.1800	0.2907	0.069*	
C19	0.3442 (3)	0.2883 (3)	0.2278 (2)	0.0527 (8)	
H19	0.3448	0.3182	0.2744	0.063*	
C20	0.6714 (3)	0.3869 (2)	0.1234 (2)	0.0479 (7)	
C21A	0.7603 (11)	0.3477 (10)	0.0654 (9)	0.077 (4)	0.50
H21A	0.7614	0.3533	0.0092	0.092*	0.50
C22A	0.8484 (13)	0.2994 (10)	0.0937 (8)	0.082 (4)	0.50
H22A	0.9092	0.2732	0.0547	0.099*	0.50
C23	0.8512 (4)	0.2881 (3)	0.1755 (3)	0.0635 (10)	
C24A	0.7924 (11)	0.3567 (9)	0.2204 (10)	0.070 (5)	0.50
H24A	0.8114	0.3701	0.2689	0.084*	0.50
C25A	0.7054 (12)	0.4056 (10)	0.1935 (11)	0.070 (4)	0.50
H25A	0.6678	0.4531	0.2236	0.084*	0.50
C26	0.9501 (5)	0.2362 (3)	0.2036 (4)	0.0882 (15)	
H26A	1.0169	0.2312	0.1570	0.106*	
H26B	0.9245	0.1769	0.2263	0.106*	
H26C	0.9715	0.2674	0.2454	0.106*	
Cl5A	0.5808 (14)	0.1138 (12)	0.3444 (10)	0.103 (4)	0.50
Cl6A	0.6252 (9)	0.0856 (5)	0.0323 (5)	0.101 (2)	0.50
S3	0.43046 (9)	−0.07138 (7)	0.40080 (6)	0.0597 (3)	
O5	0.5301 (3)	−0.0893 (2)	0.43401 (17)	0.0743 (8)	
O6	0.3574 (3)	−0.14399 (19)	0.3980 (2)	0.0750 (8)	
N3	0.3536 (3)	0.0045 (2)	0.45511 (19)	0.0612 (8)	
H3N	0.387 (4)	0.027 (3)	0.485 (3)	0.073*	
C27	0.4811 (4)	−0.0252 (3)	0.2966 (2)	0.0573 (9)	
C28A	0.546 (2)	0.0477 (16)	0.2742 (6)	0.072 (9)	0.50
C29A	0.6004 (18)	0.0809 (14)	0.1929 (9)	0.087 (9)	0.50
H29A	0.6576	0.1245	0.1806	0.105*	0.50
C30A	0.5608 (19)	0.0425 (15)	0.1328 (10)	0.079 (6)	0.50
C31A	0.503 (3)	−0.036 (2)	0.1623 (14)	0.089 (8)	0.50
H31A	0.5071	−0.0760	0.1215	0.106*	0.50
C32A	0.4397 (12)	−0.0679 (15)	0.2396 (12)	0.064 (4)	0.50
H32A	0.3806	−0.1100	0.2514	0.077*	0.50
C33	0.2459 (3)	0.0463 (3)	0.4465 (2)	0.0556 (9)	
C34	0.1758 (4)	0.0130 (3)	0.4031 (2)	0.0619 (9)	
H34	0.1994	−0.0387	0.3748	0.074*	
C35	0.0693 (4)	0.0561 (3)	0.4009 (3)	0.0668 (10)	
H35	0.0225	0.0326	0.3709	0.080*	
C36	0.0308 (4)	0.1329 (3)	0.4420 (3)	0.0663 (11)	
C37	0.1042 (4)	0.1656 (3)	0.4840 (3)	0.0754 (12)	
H37	0.0812	0.2178	0.5115	0.090*	
C38	0.2099 (4)	0.1246 (3)	0.4870 (3)	0.0698 (11)	
H38	0.2574	0.1490	0.5160	0.084*	
C39	−0.0857 (5)	0.1768 (4)	0.4424 (4)	0.0963 (17)	
H39A	−0.1465	0.1339	0.4642	0.116*	
H39B	−0.0874	0.1980	0.3866	0.116*	
H39C	−0.0986	0.2269	0.4766	0.116*	
Cl7	0.40993 (13)	0.36517 (9)	0.77244 (10)	0.0987 (5)	
Cl8	0.11054 (11)	0.15622 (9)	0.99354 (7)	0.0817 (4)	
S4	0.09578 (9)	0.02646 (6)	0.85004 (7)	0.0648 (3)	
O7	0.1111 (3)	−0.0097 (2)	0.7718 (2)	0.0848 (10)	
O8	0.1202 (3)	−0.0299 (2)	0.9177 (2)	0.0829 (10)	
N4	−0.0385 (3)	0.0638 (2)	0.8824 (2)	0.0614 (8)	
H4N	−0.059 (4)	0.057 (3)	0.9359 (17)	0.074*	
C40	0.1842 (3)	0.1232 (2)	0.8301 (2)	0.0529 (8)	
C41	0.1893 (3)	0.1785 (3)	0.8918 (2)	0.0538 (8)	
C42	0.2583 (3)	0.2528 (3)	0.8738 (3)	0.0597 (9)	
H42	0.2622	0.2893	0.9155	0.072*	
C43	0.3211 (3)	0.2724 (3)	0.7939 (3)	0.0610 (10)	
C44	0.3171 (3)	0.2202 (3)	0.7304 (3)	0.0629 (10)	
H44	0.3600	0.2348	0.6761	0.076*	
C45	0.2471 (3)	0.1451 (3)	0.7498 (3)	0.0595 (9)	
H45	0.2427	0.1089	0.7080	0.071*	
C46	−0.0905 (3)	0.1327 (3)	0.8388 (3)	0.0568 (9)	
C47	−0.0740 (4)	0.1382 (3)	0.7535 (3)	0.0723 (11)	
H47	−0.0244	0.0968	0.7215	0.087*	
C48	−0.1319 (4)	0.2055 (3)	0.7165 (3)	0.0726 (11)	
H48	−0.1188	0.2089	0.6590	0.087*	
C49	−0.2076 (4)	0.2678 (3)	0.7594 (3)	0.0654 (10)	
C50	−0.2229 (4)	0.2607 (4)	0.8440 (3)	0.0838 (14)	
H50	−0.2744	0.3014	0.8755	0.101*	
C51	−0.1645 (4)	0.1951 (4)	0.8846 (3)	0.0757 (12)	
H51	−0.1752	0.1934	0.9420	0.091*	
C52	−0.2700 (5)	0.3396 (4)	0.7162 (4)	0.0911 (15)	
H52A	−0.3174	0.3117	0.6867	0.109*	
H52B	−0.2131	0.3767	0.6776	0.109*	
H52C	−0.3193	0.3763	0.7566	0.109*	
C21B	0.7292 (11)	0.3161 (7)	0.0817 (8)	0.062 (3)	0.50
H21B	0.7096	0.3016	0.0338	0.075*	0.50
C22B	0.8162 (13)	0.2652 (8)	0.1088 (8)	0.067 (3)	0.50
H22B	0.8506	0.2151	0.0812	0.081*	0.50
C24B	0.7599 (11)	0.3289 (10)	0.2294 (10)	0.073 (4)	0.50
H24B	0.7581	0.3221	0.2857	0.088*	0.50
C25B	0.6708 (13)	0.3790 (11)	0.2065 (11)	0.071 (4)	0.50
H25B	0.6119	0.4067	0.2457	0.085*	0.50
C28B	0.555 (2)	0.0503 (14)	0.2758 (7)	0.065 (8)	0.50
C29B	0.5912 (15)	0.0761 (14)	0.1903 (7)	0.079 (7)	0.50
H29B	0.6237	0.1323	0.1720	0.095*	0.50
C30B	0.5777 (16)	0.0172 (13)	0.1333 (8)	0.069 (5)	0.50
C31B	0.501 (3)	−0.0529 (19)	0.1478 (12)	0.065 (4)	0.50
H31B	0.4743	−0.0797	0.1080	0.078*	0.50
C32B	0.4721 (12)	−0.0758 (14)	0.2320 (9)	0.052 (3)	0.50
H32B	0.4422	−0.1332	0.2481	0.063*	0.50
Cl5B	0.5918 (13)	0.1076 (12)	0.3506 (10)	0.091 (3)	0.50
Cl6B	0.6282 (9)	0.0443 (5)	0.0266 (5)	0.101 (2)	0.50

### Atomic displacement parameters (Å^2^)

**Table:**

	*U*^11^	*U*^22^	*U*^33^	*U*^12^	*U*^13^	*U*^23^
Cl1	0.0967 (8)	0.0930 (8)	0.0642 (6)	0.0389 (7)	−0.0163 (6)	0.0017 (6)
Cl2	0.0935 (9)	0.1752 (16)	0.0638 (7)	0.0248 (10)	−0.0226 (6)	−0.0444 (8)
S1	0.0539 (5)	0.0632 (5)	0.0508 (5)	0.0033 (4)	−0.0129 (4)	−0.0071 (4)
O1	0.0687 (17)	0.0607 (16)	0.0695 (17)	0.0104 (13)	−0.0153 (14)	−0.0095 (13)
O2	0.0606 (16)	0.0834 (19)	0.0607 (16)	−0.0066 (14)	−0.0182 (13)	−0.0087 (14)
N1	0.0495 (17)	0.079 (2)	0.0542 (18)	0.0019 (15)	−0.0143 (14)	−0.0013 (16)
C1	0.0480 (18)	0.062 (2)	0.054 (2)	0.0017 (16)	−0.0155 (16)	−0.0046 (16)
C2	0.053 (2)	0.069 (2)	0.057 (2)	0.0039 (17)	−0.0191 (17)	−0.0018 (18)
C3	0.052 (2)	0.082 (3)	0.074 (3)	0.009 (2)	−0.0128 (19)	−0.023 (2)
C4	0.059 (2)	0.100 (3)	0.061 (2)	0.005 (2)	−0.0171 (19)	−0.021 (2)
C5	0.080 (3)	0.100 (3)	0.057 (2)	0.012 (3)	−0.031 (2)	−0.003 (2)
C6	0.068 (2)	0.076 (3)	0.062 (2)	0.012 (2)	−0.022 (2)	−0.006 (2)
C7	0.0542 (19)	0.057 (2)	0.0438 (17)	0.0082 (16)	−0.0043 (15)	−0.0086 (15)
C8	0.078 (3)	0.061 (2)	0.069 (3)	0.012 (2)	−0.023 (2)	0.002 (2)
C9	0.093 (3)	0.049 (2)	0.081 (3)	−0.002 (2)	−0.021 (3)	0.002 (2)
C10	0.070 (2)	0.063 (2)	0.053 (2)	−0.0071 (19)	−0.0078 (18)	−0.0127 (17)
C11	0.060 (2)	0.067 (2)	0.057 (2)	0.0023 (18)	−0.0169 (18)	−0.0022 (18)
C12	0.060 (2)	0.057 (2)	0.060 (2)	−0.0021 (17)	−0.0172 (18)	0.0043 (17)
C13	0.090 (3)	0.089 (3)	0.090 (4)	−0.026 (3)	−0.016 (3)	−0.016 (3)
Cl3	0.0841 (7)	0.0865 (7)	0.0462 (5)	−0.0316 (6)	−0.0080 (4)	0.0011 (4)
Cl4	0.0800 (7)	0.0603 (6)	0.0840 (7)	−0.0256 (5)	−0.0216 (6)	0.0062 (5)
S2	0.0471 (4)	0.0448 (4)	0.0568 (5)	−0.0018 (3)	−0.0079 (4)	−0.0015 (3)
O3	0.0538 (15)	0.0551 (14)	0.0782 (18)	0.0029 (12)	−0.0128 (13)	0.0086 (13)
O4	0.0651 (16)	0.0577 (15)	0.0622 (16)	−0.0044 (12)	−0.0030 (13)	−0.0150 (12)
N2	0.0461 (15)	0.0553 (16)	0.0491 (16)	−0.0061 (13)	−0.0081 (13)	0.0025 (13)
C14	0.0416 (16)	0.0457 (17)	0.0527 (19)	−0.0020 (13)	−0.0105 (14)	−0.0005 (14)
C15	0.0486 (18)	0.057 (2)	0.0487 (18)	−0.0069 (15)	−0.0093 (15)	0.0005 (15)
C16	0.057 (2)	0.060 (2)	0.057 (2)	−0.0105 (17)	−0.0175 (17)	−0.0031 (17)
C17	0.0493 (19)	0.0492 (19)	0.065 (2)	−0.0071 (15)	−0.0159 (16)	0.0043 (16)
C18	0.058 (2)	0.058 (2)	0.053 (2)	−0.0061 (17)	−0.0100 (16)	0.0081 (16)
C19	0.0539 (19)	0.057 (2)	0.0463 (18)	−0.0040 (15)	−0.0112 (15)	0.0000 (15)
C20	0.0466 (17)	0.0426 (16)	0.0521 (19)	−0.0055 (13)	−0.0077 (14)	−0.0021 (14)
C21A	0.070 (8)	0.107 (11)	0.049 (6)	0.004 (7)	−0.004 (5)	−0.021 (7)
C22A	0.065 (8)	0.095 (11)	0.090 (8)	0.027 (7)	−0.019 (6)	−0.042 (8)
C23	0.067 (2)	0.055 (2)	0.072 (3)	0.0029 (18)	−0.024 (2)	−0.0080 (19)
C24A	0.063 (7)	0.085 (9)	0.075 (9)	0.013 (7)	−0.036 (6)	−0.033 (8)
C25A	0.060 (8)	0.077 (8)	0.078 (10)	0.008 (6)	−0.013 (7)	−0.043 (7)
C26	0.096 (4)	0.069 (3)	0.113 (4)	0.023 (3)	−0.050 (3)	−0.017 (3)
Cl5A	0.147 (9)	0.086 (4)	0.070 (4)	−0.018 (4)	−0.009 (4)	−0.025 (3)
Cl6A	0.089 (2)	0.157 (6)	0.050 (2)	0.004 (4)	−0.0106 (16)	0.014 (3)
S3	0.0698 (6)	0.0593 (5)	0.0463 (5)	0.0149 (4)	−0.0109 (4)	−0.0049 (4)
O5	0.084 (2)	0.084 (2)	0.0546 (16)	0.0286 (16)	−0.0223 (15)	−0.0103 (14)
O6	0.088 (2)	0.0569 (16)	0.0748 (19)	0.0049 (15)	−0.0128 (16)	−0.0022 (14)
N3	0.065 (2)	0.074 (2)	0.0438 (16)	0.0119 (16)	−0.0105 (14)	−0.0147 (15)
C27	0.058 (2)	0.066 (2)	0.046 (2)	0.017 (2)	−0.0119 (17)	−0.0069 (17)
C28A	0.076 (14)	0.099 (19)	0.044 (11)	0.030 (11)	−0.018 (9)	−0.028 (10)
C29A	0.082 (12)	0.093 (13)	0.093 (16)	0.019 (9)	−0.046 (10)	0.024 (10)
C30A	0.050 (9)	0.116 (13)	0.057 (7)	0.043 (8)	−0.009 (5)	0.025 (7)
C31A	0.086 (11)	0.142 (17)	0.049 (10)	0.029 (11)	−0.033 (9)	−0.042 (11)
C32A	0.021 (7)	0.098 (9)	0.067 (7)	0.026 (6)	0.001 (5)	−0.023 (5)
C33	0.059 (2)	0.055 (2)	0.0456 (18)	0.0024 (16)	−0.0003 (16)	−0.0022 (15)
C34	0.072 (2)	0.055 (2)	0.055 (2)	0.0047 (18)	−0.0071 (18)	−0.0116 (17)
C35	0.065 (2)	0.065 (2)	0.066 (2)	−0.0008 (19)	−0.0099 (19)	−0.0027 (19)
C36	0.070 (3)	0.059 (2)	0.057 (2)	0.0066 (19)	0.0019 (19)	0.0057 (18)
C37	0.082 (3)	0.059 (2)	0.077 (3)	0.013 (2)	−0.004 (2)	−0.019 (2)
C38	0.079 (3)	0.063 (2)	0.065 (2)	0.002 (2)	−0.009 (2)	−0.0191 (19)
C39	0.074 (3)	0.091 (4)	0.105 (4)	0.021 (3)	0.004 (3)	0.003 (3)
Cl7	0.0976 (9)	0.0855 (8)	0.1174 (10)	−0.0483 (7)	−0.0412 (8)	0.0371 (7)
Cl8	0.0762 (7)	0.1067 (9)	0.0576 (6)	−0.0272 (6)	−0.0043 (5)	−0.0050 (5)
S4	0.0627 (6)	0.0440 (5)	0.0844 (7)	−0.0090 (4)	−0.0124 (5)	−0.0004 (4)
O7	0.091 (2)	0.0576 (17)	0.104 (3)	−0.0044 (16)	−0.0139 (19)	−0.0260 (17)
O8	0.0742 (19)	0.0536 (16)	0.113 (3)	−0.0054 (14)	−0.0187 (18)	0.0211 (16)
N4	0.0540 (18)	0.0605 (18)	0.067 (2)	−0.0127 (14)	−0.0109 (16)	0.0021 (16)
C40	0.0480 (18)	0.0441 (17)	0.063 (2)	−0.0017 (14)	−0.0094 (16)	0.0009 (15)
C41	0.0443 (18)	0.056 (2)	0.059 (2)	−0.0050 (15)	−0.0094 (15)	0.0009 (16)
C42	0.054 (2)	0.057 (2)	0.071 (2)	−0.0050 (17)	−0.0201 (18)	−0.0024 (18)
C43	0.0454 (19)	0.055 (2)	0.081 (3)	−0.0094 (16)	−0.0191 (18)	0.0176 (19)
C44	0.0485 (19)	0.068 (2)	0.064 (2)	0.0012 (17)	−0.0050 (17)	0.0138 (19)
C45	0.057 (2)	0.056 (2)	0.061 (2)	0.0063 (17)	−0.0085 (17)	−0.0060 (17)
C46	0.0447 (18)	0.055 (2)	0.069 (2)	−0.0149 (15)	−0.0070 (16)	−0.0100 (17)
C47	0.085 (3)	0.062 (2)	0.069 (3)	0.003 (2)	−0.012 (2)	−0.018 (2)
C48	0.084 (3)	0.074 (3)	0.057 (2)	−0.003 (2)	−0.011 (2)	−0.011 (2)
C49	0.052 (2)	0.075 (3)	0.066 (2)	−0.0033 (18)	−0.0064 (18)	−0.007 (2)
C50	0.068 (3)	0.102 (4)	0.074 (3)	0.021 (3)	−0.004 (2)	−0.019 (3)
C51	0.067 (3)	0.099 (3)	0.054 (2)	0.004 (2)	−0.0002 (19)	−0.015 (2)
C52	0.078 (3)	0.095 (4)	0.093 (4)	0.011 (3)	−0.015 (3)	0.003 (3)
C21B	0.064 (7)	0.073 (7)	0.054 (7)	0.006 (5)	−0.018 (5)	−0.024 (5)
C22B	0.069 (8)	0.056 (6)	0.088 (8)	0.010 (5)	−0.032 (6)	−0.033 (5)
C24B	0.070 (8)	0.099 (10)	0.051 (5)	0.003 (7)	−0.019 (6)	−0.003 (6)
C25B	0.058 (8)	0.097 (11)	0.054 (6)	0.019 (7)	−0.014 (6)	−0.006 (7)
C28B	0.051 (9)	0.058 (11)	0.075 (15)	−0.004 (8)	0.002 (8)	0.010 (8)
C29B	0.051 (8)	0.117 (15)	0.053 (10)	−0.015 (8)	0.021 (6)	−0.016 (8)
C30B	0.040 (6)	0.124 (14)	0.031 (5)	0.043 (9)	−0.005 (4)	0.010 (6)
C31B	0.060 (7)	0.115 (11)	0.026 (6)	0.007 (6)	−0.014 (5)	−0.031 (7)
C32B	0.026 (8)	0.086 (6)	0.042 (7)	0.033 (6)	−0.006 (5)	−0.021 (4)
Cl5B	0.095 (4)	0.100 (5)	0.076 (4)	−0.028 (3)	−0.010 (3)	−0.012 (3)
Cl6B	0.080 (2)	0.174 (6)	0.0448 (16)	−0.013 (4)	−0.0067 (14)	0.004 (4)

### Geometric parameters (Å, °)

**Table:**

Cl1—C2	1.731 (4)	N3—H3N	0.82 (3)
Cl2—C4	1.730 (4)	C27—C28A	1.327 (19)
S1—O1	1.421 (3)	C27—C32A	1.384 (13)
S1—O2	1.434 (3)	C27—C32B	1.394 (11)
S1—N1	1.600 (4)	C27—C28B	1.420 (15)
S1—C1	1.773 (4)	C28A—C29A	1.403 (14)
N1—C7	1.433 (5)	C29A—C30A	1.382 (15)
N1—H1N	0.83 (3)	C29A—H29A	0.9300
C1—C6	1.379 (6)	C30A—C31A	1.372 (16)
C1—C2	1.381 (5)	C31A—C32A	1.370 (15)
C2—C3	1.382 (6)	C31A—H31A	0.9300
C3—C4	1.358 (7)	C32A—H32A	0.9300
C3—H3	0.9300	C33—C34	1.361 (6)
C4—C5	1.371 (7)	C33—C38	1.392 (5)
C5—C6	1.381 (6)	C34—C35	1.386 (6)
C5—H5	0.9300	C34—H34	0.9300
C6—H6	0.9300	C35—C36	1.380 (6)
C7—C12	1.365 (5)	C35—H35	0.9300
C7—C8	1.373 (6)	C36—C37	1.371 (7)
C8—C9	1.382 (7)	C36—C39	1.488 (6)
C8—H8	0.9300	C37—C38	1.367 (7)
C9—C10	1.372 (6)	C37—H37	0.9300
C9—H9	0.9300	C38—H38	0.9300
C10—C11	1.369 (6)	C39—H39A	0.9600
C10—C13	1.525 (6)	C39—H39B	0.9600
C11—C12	1.387 (6)	C39—H39C	0.9600
C11—H11	0.9300	Cl7—C43	1.729 (4)
C12—H12	0.9300	Cl8—C41	1.726 (4)
C13—H13A	0.9600	S4—O7	1.419 (4)
C13—H13B	0.9600	S4—O8	1.427 (3)
C13—H13C	0.9600	S4—N4	1.620 (4)
Cl3—C15	1.727 (4)	S4—C40	1.775 (4)
Cl4—C17	1.722 (4)	N4—C46	1.411 (5)
S2—O4	1.417 (3)	N4—H4N	0.86 (3)
S2—O3	1.426 (3)	C40—C45	1.374 (5)
S2—N2	1.609 (3)	C40—C41	1.386 (5)
S2—C14	1.782 (3)	C41—C42	1.369 (5)
N2—C20	1.423 (5)	C42—C43	1.364 (6)
N2—H2N	0.85 (3)	C42—H42	0.9300
C14—C15	1.383 (5)	C43—C44	1.378 (6)
C14—C19	1.386 (5)	C44—C45	1.386 (6)
C15—C16	1.380 (5)	C44—H44	0.9300
C16—C17	1.382 (5)	C45—H45	0.9300
C16—H16	0.9300	C46—C51	1.381 (6)
C17—C18	1.365 (6)	C46—C47	1.382 (6)
C18—C19	1.388 (5)	C47—C48	1.373 (7)
C18—H18	0.9300	C47—H47	0.9300
C19—H19	0.9300	C48—C49	1.368 (6)
C20—C21B	1.368 (10)	C48—H48	0.9300
C20—C21A	1.376 (11)	C49—C50	1.371 (7)
C20—C25B	1.379 (18)	C49—C52	1.503 (7)
C20—C25A	1.382 (18)	C50—C51	1.393 (7)
C21A—C22A	1.389 (13)	C50—H50	0.9300
C21A—H21A	0.9300	C51—H51	0.9300
C22A—C23	1.369 (12)	C52—H52A	0.9600
C22A—H22A	0.9300	C52—H52B	0.9600
C23—C22B	1.355 (11)	C52—H52C	0.9600
C23—C24B	1.366 (12)	C21B—C22B	1.388 (12)
C23—C24A	1.371 (11)	C21B—H21B	0.9300
C23—C26	1.515 (6)	C22B—H22B	0.9300
C24A—C25A	1.372 (13)	C24B—C25B	1.371 (12)
C24A—H24A	0.9300	C24B—H24B	0.9300
C25A—H25A	0.9300	C25B—H25B	0.9300
C26—H26A	0.9600	C28B—C29B	1.404 (13)
C26—H26B	0.9600	C28B—Cl5B	1.721 (12)
C26—H26C	0.9600	C29B—C30B	1.390 (14)
Cl5A—C28A	1.728 (12)	C29B—H29B	0.9300
Cl6A—C30A	1.734 (12)	C30B—C31B	1.375 (16)
S3—O5	1.423 (3)	C30B—Cl6B	1.744 (10)
S3—O6	1.424 (3)	C31B—C32B	1.375 (14)
S3—N3	1.603 (3)	C31B—H31B	0.9300
S3—C27	1.775 (4)	C32B—H32B	0.9300
N3—C33	1.419 (5)		
			
O1—S1—O2	119.00 (19)	C28A—C27—C32B	115.8 (10)
O1—S1—N1	109.73 (18)	C32A—C27—C32B	16.0 (9)
O2—S1—N1	105.10 (18)	C28A—C27—C28B	4(2)
O1—S1—C1	106.05 (18)	C32A—C27—C28B	124.1 (12)
O2—S1—C1	108.63 (17)	C32B—C27—C28B	117.5 (10)
N1—S1—C1	107.94 (19)	C28A—C27—S3	124.3 (6)
C7—N1—S1	127.5 (3)	C32A—C27—S3	114.0 (11)
C7—N1—H1N	120 (3)	C32B—C27—S3	118.9 (9)
S1—N1—H1N	107 (3)	C28B—C27—S3	121.8 (6)
C6—C1—C2	117.9 (4)	C27—C28A—C29A	126.8 (15)
C6—C1—S1	118.5 (3)	C27—C28A—Cl5A	123.5 (9)
C2—C1—S1	123.5 (3)	C29A—C28A—Cl5A	109.6 (18)
C1—C2—C3	120.9 (4)	C30A—C29A—C28A	113.4 (17)
C1—C2—Cl1	121.7 (3)	C30A—C29A—H29A	123.3
C3—C2—Cl1	117.4 (3)	C28A—C29A—H29A	123.3
C4—C3—C2	119.6 (4)	C31A—C30A—C29A	112.6 (17)
C4—C3—H3	120.2	C31A—C30A—Cl6A	131.5 (19)
C2—C3—H3	120.2	C29A—C30A—Cl6A	113.5 (14)
C3—C4—C5	121.3 (4)	C32A—C31A—C30A	133 (2)
C3—C4—Cl2	118.5 (4)	C32A—C31A—H31A	113.4
C5—C4—Cl2	120.2 (4)	C30A—C31A—H31A	113.4
C4—C5—C6	118.4 (4)	C31A—C32A—C27	106.5 (17)
C4—C5—H5	120.8	C31A—C32A—H32A	126.7
C6—C5—H5	120.8	C27—C32A—H32A	126.7
C1—C6—C5	121.9 (4)	C34—C33—C38	119.0 (4)
C1—C6—H6	119.1	C34—C33—N3	123.9 (3)
C5—C6—H6	119.1	C38—C33—N3	117.1 (4)
C12—C7—C8	119.8 (4)	C33—C34—C35	120.0 (4)
C12—C7—N1	123.3 (4)	C33—C34—H34	120.0
C8—C7—N1	116.9 (4)	C35—C34—H34	120.0
C7—C8—C9	119.9 (4)	C36—C35—C34	121.9 (4)
C7—C8—H8	120.1	C36—C35—H35	119.1
C9—C8—H8	120.1	C34—C35—H35	119.1
C10—C9—C8	121.2 (4)	C37—C36—C35	116.7 (4)
C10—C9—H9	119.4	C37—C36—C39	121.6 (4)
C8—C9—H9	119.4	C35—C36—C39	121.7 (5)
C11—C10—C9	118.0 (4)	C38—C37—C36	122.7 (4)
C11—C10—C13	121.4 (4)	C38—C37—H37	118.6
C9—C10—C13	120.6 (4)	C36—C37—H37	118.6
C10—C11—C12	121.6 (4)	C37—C38—C33	119.6 (4)
C10—C11—H11	119.2	C37—C38—H38	120.2
C12—C11—H11	119.2	C33—C38—H38	120.2
C7—C12—C11	119.5 (4)	C36—C39—H39A	109.5
C7—C12—H12	120.2	C36—C39—H39B	109.5
C11—C12—H12	120.2	H39A—C39—H39B	109.5
C10—C13—H13A	109.5	C36—C39—H39C	109.5
C10—C13—H13B	109.5	H39A—C39—H39C	109.5
H13A—C13—H13B	109.5	H39B—C39—H39C	109.5
C10—C13—H13C	109.5	O7—S4—O8	119.1 (2)
H13A—C13—H13C	109.5	O7—S4—N4	109.8 (2)
H13B—C13—H13C	109.5	O8—S4—N4	106.0 (2)
O4—S2—O3	119.20 (17)	O7—S4—C40	105.50 (19)
O4—S2—N2	109.43 (17)	O8—S4—C40	110.06 (19)
O3—S2—N2	105.88 (16)	N4—S4—C40	105.68 (17)
O4—S2—C14	105.53 (16)	C46—N4—S4	123.7 (3)
O3—S2—C14	108.17 (17)	C46—N4—H4N	121 (3)
N2—S2—C14	108.26 (16)	S4—N4—H4N	109 (3)
C20—N2—S2	123.1 (2)	C45—C40—C41	119.1 (3)
C20—N2—H2N	116 (3)	C45—C40—S4	118.4 (3)
S2—N2—H2N	116 (3)	C41—C40—S4	122.5 (3)
C15—C14—C19	118.7 (3)	C42—C41—C40	120.7 (4)
C15—C14—S2	123.5 (3)	C42—C41—Cl8	117.7 (3)
C19—C14—S2	117.6 (3)	C40—C41—Cl8	121.6 (3)
C16—C15—C14	120.8 (3)	C43—C42—C41	119.1 (4)
C16—C15—Cl3	117.7 (3)	C43—C42—H42	120.5
C14—C15—Cl3	121.5 (3)	C41—C42—H42	120.5
C15—C16—C17	119.0 (4)	C42—C43—C44	122.2 (4)
C15—C16—H16	120.5	C42—C43—Cl7	118.6 (3)
C17—C16—H16	120.5	C44—C43—Cl7	119.3 (3)
C18—C17—C16	121.6 (3)	C43—C44—C45	117.9 (4)
C18—C17—Cl4	120.4 (3)	C43—C44—H44	121.0
C16—C17—Cl4	118.0 (3)	C45—C44—H44	121.0
C17—C18—C19	118.7 (3)	C40—C45—C44	121.0 (4)
C17—C18—H18	120.6	C40—C45—H45	119.5
C19—C18—H18	120.6	C44—C45—H45	119.5
C14—C19—C18	121.0 (3)	C51—C46—C47	119.2 (4)
C14—C19—H19	119.5	C51—C46—N4	117.8 (4)
C18—C19—H19	119.5	C47—C46—N4	123.0 (4)
C21B—C20—C21A	25.7 (7)	C48—C47—C46	119.0 (4)
C21B—C20—C25B	112.6 (9)	C48—C47—H47	120.5
C21A—C20—C25B	120.9 (10)	C46—C47—H47	120.5
C21B—C20—C25A	116.3 (9)	C49—C48—C47	123.9 (4)
C21A—C20—C25A	113.0 (9)	C49—C48—H48	118.1
C25B—C20—C25A	23.9 (8)	C47—C48—H48	118.1
C21B—C20—N2	121.4 (7)	C48—C49—C50	116.0 (4)
C21A—C20—N2	117.7 (8)	C48—C49—C52	121.9 (4)
C25B—C20—N2	121.3 (7)	C50—C49—C52	122.1 (4)
C25A—C20—N2	122.3 (7)	C49—C50—C51	122.6 (4)
C20—C21A—C22A	117.6 (13)	C49—C50—H50	118.7
C20—C21A—H21A	121.2	C51—C50—H50	118.7
C22A—C21A—H21A	121.2	C46—C51—C50	119.3 (4)
C23—C22A—C21A	124.0 (13)	C46—C51—H51	120.4
C23—C22A—H22A	118.0	C50—C51—H51	120.4
C21A—C22A—H22A	118.0	C49—C52—H52A	109.5
C22B—C23—C24B	110.0 (10)	C49—C52—H52B	109.5
C22B—C23—C22A	27.4 (7)	H52A—C52—H52B	109.5
C24B—C23—C22A	115.0 (10)	C49—C52—H52C	109.5
C22B—C23—C24A	119.2 (10)	H52A—C52—H52C	109.5
C24B—C23—C24A	23.7 (9)	H52B—C52—H52C	109.5
C22A—C23—C24A	112.2 (10)	C20—C21B—C22B	121.9 (12)
C22B—C23—C26	121.0 (7)	C20—C21B—H21B	119.1
C24B—C23—C26	122.7 (8)	C22B—C21B—H21B	119.1
C22A—C23—C26	122.2 (7)	C23—C22B—C21B	120.2 (12)
C24A—C23—C26	119.7 (8)	C23—C22B—H22B	119.9
C23—C24A—C25A	119.7 (13)	C21B—C22B—H22B	119.9
C23—C24A—H24A	120.2	C23—C24B—C25B	124.7 (14)
C25A—C24A—H24A	120.2	C23—C24B—H24B	117.6
C24A—C25A—C20	122.5 (13)	C25B—C24B—H24B	117.6
C24A—C25A—H25A	118.7	C24B—C25B—C20	117.8 (15)
C20—C25A—H25A	118.7	C24B—C25B—H25B	121.1
C23—C26—H26A	109.5	C20—C25B—H25B	121.1
C23—C26—H26B	109.5	C29B—C28B—C27	114.7 (14)
H26A—C26—H26B	109.5	C29B—C28B—Cl5B	123.3 (15)
C23—C26—H26C	109.5	C27—C28B—Cl5B	121.9 (8)
H26A—C26—H26C	109.5	C30B—C29B—C28B	119.5 (15)
H26B—C26—H26C	109.5	C30B—C29B—H29B	120.3
O5—S3—O6	118.92 (19)	C28B—C29B—H29B	120.3
O5—S3—N3	105.43 (18)	C31B—C30B—C29B	126.9 (13)
O6—S3—N3	109.4 (2)	C31B—C30B—Cl6B	110.0 (14)
O5—S3—C27	107.92 (19)	C29B—C30B—Cl6B	120.9 (12)
O6—S3—C27	106.1 (2)	C30B—C31B—C32B	107.2 (18)
N3—S3—C27	108.71 (18)	C30B—C31B—H31B	126.4
C33—N3—S3	127.8 (3)	C32B—C31B—H31B	126.4
C33—N3—H3N	117 (4)	C31B—C32B—C27	129.0 (18)
S3—N3—H3N	114 (4)	C31B—C32B—H32B	115.5
C28A—C27—C32A	121.6 (12)	C27—C32B—H32B	115.5
			
O1—S1—N1—C7	−54.5 (4)	C32B—C27—C28A—Cl5A	−173.3 (18)
O2—S1—N1—C7	176.4 (3)	C28B—C27—C28A—Cl5A	−56 (23)
C1—S1—N1—C7	60.6 (4)	S3—C27—C28A—Cl5A	−5(3)
O1—S1—C1—C6	−6.6 (4)	C27—C28A—C29A—C30A	14 (4)
O2—S1—C1—C6	122.4 (3)	Cl5A—C28A—C29A—C30A	−169.1 (17)
N1—S1—C1—C6	−124.2 (3)	C28A—C29A—C30A—C31A	−17 (3)
O1—S1—C1—C2	176.5 (3)	C28A—C29A—C30A—Cl6A	178.3 (17)
O2—S1—C1—C2	−54.5 (4)	C29A—C30A—C31A—C32A	28 (5)
N1—S1—C1—C2	59.0 (4)	Cl6A—C30A—C31A—C32A	−171 (3)
C6—C1—C2—C3	0.9 (6)	C30A—C31A—C32A—C27	−25 (5)
S1—C1—C2—C3	177.8 (3)	C28A—C27—C32A—C31A	16 (3)
C6—C1—C2—Cl1	−179.4 (3)	C32B—C27—C32A—C31A	−57 (6)
S1—C1—C2—Cl1	−2.5 (5)	C28B—C27—C32A—C31A	12 (3)
C1—C2—C3—C4	−0.7 (7)	S3—C27—C32A—C31A	−169 (2)
Cl1—C2—C3—C4	179.6 (4)	S3—N3—C33—C34	16.6 (6)
C2—C3—C4—C5	0.7 (7)	S3—N3—C33—C38	−165.1 (3)
C2—C3—C4—Cl2	−179.5 (3)	C38—C33—C34—C35	−1.1 (6)
C3—C4—C5—C6	−0.9 (8)	N3—C33—C34—C35	177.1 (4)
Cl2—C4—C5—C6	179.4 (4)	C33—C34—C35—C36	−0.1 (6)
C2—C1—C6—C5	−1.1 (7)	C34—C35—C36—C37	1.2 (6)
S1—C1—C6—C5	−178.2 (4)	C34—C35—C36—C39	−177.5 (4)
C4—C5—C6—C1	1.1 (7)	C35—C36—C37—C38	−1.0 (7)
S1—N1—C7—C12	28.6 (5)	C39—C36—C37—C38	177.6 (5)
S1—N1—C7—C8	−154.0 (3)	C36—C37—C38—C33	−0.2 (7)
C12—C7—C8—C9	0.5 (6)	C34—C33—C38—C37	1.3 (6)
N1—C7—C8—C9	−177.0 (4)	N3—C33—C38—C37	−177.1 (4)
C7—C8—C9—C10	−0.7 (7)	O7—S4—N4—C46	−60.3 (4)
C8—C9—C10—C11	0.5 (7)	O8—S4—N4—C46	169.8 (3)
C8—C9—C10—C13	178.8 (4)	C40—S4—N4—C46	53.0 (4)
C9—C10—C11—C12	−0.2 (6)	O7—S4—C40—C45	−0.1 (4)
C13—C10—C11—C12	−178.4 (4)	O8—S4—C40—C45	129.6 (3)
C8—C7—C12—C11	−0.2 (6)	N4—S4—C40—C45	−116.5 (3)
N1—C7—C12—C11	177.1 (4)	O7—S4—C40—C41	178.1 (3)
C10—C11—C12—C7	0.1 (6)	O8—S4—C40—C41	−52.3 (4)
O4—S2—N2—C20	54.9 (3)	N4—S4—C40—C41	61.7 (4)
O3—S2—N2—C20	−175.5 (3)	C45—C40—C41—C42	−1.6 (6)
C14—S2—N2—C20	−59.7 (3)	S4—C40—C41—C42	−179.8 (3)
O4—S2—C14—C15	−178.0 (3)	C45—C40—C41—Cl8	178.9 (3)
O3—S2—C14—C15	53.4 (3)	S4—C40—C41—Cl8	0.7 (5)
N2—S2—C14—C15	−60.9 (3)	C40—C41—C42—C43	0.8 (6)
O4—S2—C14—C19	7.4 (3)	Cl8—C41—C42—C43	−179.7 (3)
O3—S2—C14—C19	−121.2 (3)	C41—C42—C43—C44	0.3 (6)
N2—S2—C14—C19	124.5 (3)	C41—C42—C43—Cl7	−178.8 (3)
C19—C14—C15—C16	1.5 (5)	C42—C43—C44—C45	−0.6 (6)
S2—C14—C15—C16	−173.1 (3)	Cl7—C43—C44—C45	178.6 (3)
C19—C14—C15—Cl3	−178.3 (3)	C41—C40—C45—C44	1.4 (6)
S2—C14—C15—Cl3	7.1 (5)	S4—C40—C45—C44	179.6 (3)
C14—C15—C16—C17	0.9 (6)	C43—C44—C45—C40	−0.3 (6)
Cl3—C15—C16—C17	−179.3 (3)	S4—N4—C46—C51	−141.1 (4)
C15—C16—C17—C18	−2.5 (6)	S4—N4—C46—C47	41.4 (5)
C15—C16—C17—Cl4	176.5 (3)	C51—C46—C47—C48	−0.1 (6)
C16—C17—C18—C19	1.7 (6)	N4—C46—C47—C48	177.4 (4)
Cl4—C17—C18—C19	−177.3 (3)	C46—C47—C48—C49	−1.1 (7)
C15—C14—C19—C18	−2.3 (5)	C47—C48—C49—C50	0.8 (7)
S2—C14—C19—C18	172.5 (3)	C47—C48—C49—C52	−179.3 (5)
C17—C18—C19—C14	0.8 (6)	C48—C49—C50—C51	0.8 (8)
S2—N2—C20—C21B	112.0 (7)	C52—C49—C50—C51	−179.2 (5)
S2—N2—C20—C21A	141.3 (7)	C47—C46—C51—C50	1.5 (7)
S2—N2—C20—C25B	−41.9 (8)	N4—C46—C51—C50	−176.1 (4)
S2—N2—C20—C25A	−70.1 (8)	C49—C50—C51—C46	−1.9 (8)
C21B—C20—C21A—C22A	−77 (3)	C21A—C20—C21B—C22B	89 (3)
C25B—C20—C21A—C22A	0.5 (18)	C25B—C20—C21B—C22B	−25.2 (15)
C25A—C20—C21A—C22A	25.8 (16)	C25A—C20—C21B—C22B	0.7 (16)
N2—C20—C21A—C22A	177.3 (10)	N2—C20—C21B—C22B	178.8 (9)
C20—C21A—C22A—C23	1(2)	C24B—C23—C22B—C21B	30.1 (16)
C21A—C22A—C23—C22B	85 (3)	C22A—C23—C22B—C21B	−76 (2)
C21A—C22A—C23—C24B	−0.5 (19)	C24A—C23—C22B—C21B	6.1 (17)
C21A—C22A—C23—C24A	−26.2 (18)	C26—C23—C22B—C21B	−177.0 (10)
C21A—C22A—C23—C26	−179.0 (11)	C20—C21B—C22B—C23	−3.9 (19)
C22B—C23—C24A—C25A	−5.1 (18)	C22B—C23—C24B—C25B	−29.9 (18)
C24B—C23—C24A—C25A	−78 (3)	C22A—C23—C24B—C25B	−0.8 (19)
C22A—C23—C24A—C25A	24.4 (17)	C24A—C23—C24B—C25B	88 (4)
C26—C23—C24A—C25A	177.9 (11)	C26—C23—C24B—C25B	177.7 (12)
C23—C24A—C25A—C20	2(2)	C23—C24B—C25B—C20	2(2)
C21B—C20—C25A—C24A	0.3 (17)	C21B—C20—C25B—C24B	25.7 (16)
C21A—C20—C25A—C24A	−27.9 (17)	C21A—C20—C25B—C24B	−1.6 (18)
C25B—C20—C25A—C24A	87 (3)	C25A—C20—C25B—C24B	−78 (3)
N2—C20—C25A—C24A	−177.8 (11)	N2—C20—C25B—C24B	−178.3 (10)
O5—S3—N3—C33	179.4 (3)	C28A—C27—C28B—C29B	−51 (23)
O6—S3—N3—C33	−51.6 (4)	C32A—C27—C28B—C29B	−4(3)
C27—S3—N3—C33	63.9 (4)	C32B—C27—C28B—C29B	13 (3)
O5—S3—C27—C28A	−56.0 (16)	S3—C27—C28B—C29B	177.6 (14)
O6—S3—C27—C28A	175.5 (16)	C28A—C27—C28B—Cl5B	126 (27)
N3—S3—C27—C28A	57.8 (16)	C32A—C27—C28B—Cl5B	173.3 (16)
O5—S3—C27—C32A	128.8 (7)	C32B—C27—C28B—Cl5B	−169.8 (17)
O6—S3—C27—C32A	0.3 (8)	S3—C27—C28B—Cl5B	−5(3)
N3—S3—C27—C32A	−117.4 (7)	C27—C28B—C29B—C30B	−15 (3)
O5—S3—C27—C32B	111.8 (7)	Cl5B—C28B—C29B—C30B	167.6 (19)
O6—S3—C27—C32B	−16.7 (7)	C28B—C29B—C30B—C31B	22 (4)
N3—S3—C27—C32B	−134.4 (7)	C28B—C29B—C30B—Cl6B	−176.6 (18)
O5—S3—C27—C28B	−52.6 (15)	C29B—C30B—C31B—C32B	−22 (4)
O6—S3—C27—C28B	178.8 (15)	Cl6B—C30B—C31B—C32B	175.2 (19)
N3—S3—C27—C28B	61.2 (15)	C30B—C31B—C32B—C27	20 (4)
C32A—C27—C28A—C29A	−14 (4)	C28A—C27—C32B—C31B	−14 (3)
C32B—C27—C28A—C29A	3(3)	C32A—C27—C32B—C31B	101 (8)
C28B—C27—C28A—C29A	121 (27)	C28B—C27—C32B—C31B	−17 (3)
S3—C27—C28A—C29A	171.5 (19)	S3—C27—C32B—C31B	177 (2)
C32A—C27—C28A—Cl5A	169.7 (17)		

### Hydrogen-bond geometry (Å, °)

**Table:**

*D*—H···*A*	*D*—H	H···*A*	*D*···*A*	*D*—H···*A*
N1—H1N···O2^i^	0.83 (3)	2.26 (3)	3.079 (4)	169 (5)
N2—H2N···O3^ii^	0.85 (3)	2.24 (3)	3.080 (4)	169 (4)
N3—H3N···O5^iii^	0.82 (3)	2.14 (3)	2.956 (5)	178 (5)
N4—H4N···O8^iv^	0.86 (3)	2.37 (3)	3.226 (5)	177 (4)

Symmetry codes: (i) −*x*, −*y*+1, −*z*+1; (ii) −*x*+1, −*y*+1, −*z*; (iii) −*x*+1, −*y*, −*z*+1; (iv) −*x*, −*y*, −*z*+2.
